# Negotiating Meaning via Communication Strategies: EFL Learners’ Behavior in Peer Interaction

**DOI:** 10.3390/bs15070976

**Published:** 2025-07-18

**Authors:** Changying Li

**Affiliations:** School of Foreign Languages, Huazhong University of Science and Technology, Wuhan 430074, China; lichangying@hust.edu.cn

**Keywords:** communication strategies, negotiation of meaning, peer interaction

## Abstract

This study examines how Chinese EFL learners behave in peer interactions by negotiating meaning through communication strategies. This is a small-scale study with both quantitative and qualitative analysis. Sixteen Chinese EFL students and one native English teacher were observed in an intensive English program. The students were asked to interact with their peers in a decision-making task and an information-gap task. Video-recorded data were collected, transcribed and analyzed. The results showed that negotiation occur more frequently in information-gap tasks than decision-making tasks and students primarily employed confirmation checks. For communication strategies used to negotiate, direct strategies were employed most frequently, in which students mainly used code-switch and mime. Indirect strategies followed, with repetition occurring as the most frequently employed strategy. Interactional strategies, including co-construction and appeal for help, were less frequently used. The findings highlight the influence of cultural factors and students’ motivation on their behaviors.

## 1. Introduction

In the realm of second language acquisition (SLA) and applied linguistics, the study of communication strategies (CSs) is a vital area of research, offering insights into language learners’ behaviors in managing communicative deficiencies and achieving successful interaction. CSs are mutual attempts of interlocutors to solve problems in communication and keep the communication channel open when they perceive that the problem will become an obstacle in communication. These strategies not only include traditional problem-solving devices used by interlocutors to communicate in alternative way, but also those strategies that keep the communication channel open without being strictly problem-oriented (Dörnyei & Scott, 1997). The importance of communication strategies is underscored by their role in enhancing learners’ communicative competence. As defined by Canale and Swain (1980), communicative competence encompasses not only grammatical and sociolinguistic aspects but also strategic competence, which involves the ability to use various means to compensate for gaps in linguistic knowledge. This strategic competence is crucial for effective communication, as it allows learners to adapt their language use to different contexts and interlocutors. Research has shown that enhancing learners’ strategic competence is an effective and economic way to promote their communicative competence (Canale & Swain, 1980; Chen, 1990; Savignon, 1991).

Along with CSs, negotiation of meaning (NoM) also contributes to the development of communicative competence. While CSs help learners manage immediate communicative needs, NoM provides a context for learners to receive feedback and adjust their language use (Yi & Sun, 2013). This feedback loop is essential for linguistic development and the acquisition of new vocabulary and grammatical structures (Yi & Sun, 2013). NoM is essential for language acquisition as it provides learners with comprehensible input, promotes modified output and encourages language exploration and attention to form. When communication breakdowns occur, interlocutors use a range of CSs to negotiate meaning, achieve mutual understanding and move the conversation forward. CSs can initiate or facilitate NoM, while NoM can provide opportunities for learners to practice and refine their CSs. Through repeated interactions and negotiations, learners become more adept at using various strategies to manage communication challenges.

Both CSs and NoM have received considerable attention, but few studies have explored how CSs are applied within NoM to provide a comprehensive view of students’ interactional behaviors. More importantly, research should prioritize natural classroom settings over laboratory settings to ensure that findings are applicable to real-world teaching contexts. The present study aims to examine how Chinese EFL students apply CSs in NoM while communicating with their peers in an International Summer Intensive English Program.

## 2. Literature Review

### 2.1. Communication Strategies

CSs have been conceptualized from two primary perspectives: the psycholinguistic and the interactive (Zhu et al., 2024). The psycholinguistic perspective defines CSs as conscious plans to solve communication problems, focusing on learners’ cognitive processes (Færch & Kasper, 1984). In contrast, the interactive perspective views CSs as collaborative efforts of speakers to reach mutual understanding, emphasizing the dynamics of meaning-creating processes (Tarone, 1981; Zhu et al., 2024). Empirical studies tend to combine both perspectives for accurate and comprehensive identification and classification (Rosas-Maldonado, 2017). This study defines CSs broadly as any attempt by learners to settle communication breakdowns and to keep the communication channel open in order to achieve communication goals.

Different taxonomies of CSs were used by researchers. Tarone’s (1978) foundational taxonomy identified five basic CSs: avoidance (including topic and message avoidance), paraphrase (including approximation, word coinage and circumlocution), conscious transfer (including literal translation and language switch), appeal for assistance and mime. Despite its enduring influence, Færch and Kasper (1984) critiqued this typology for misalignment with Tarone’s interactional definition. They proposed a binary classification: achievement and reduction strategies. Achievement strategies enable learners to use an alternative way to reach their communication goal. Reduction strategies, however, were used by learners to avoid or give up solving communication problems (Nakatani, 2010). Integrating the previous taxonomies of CSs, Dörnyei and Scott (1997) classified CSs into three basic categories according to the manner of problem-management: direct, indirect and interactional strategies. Direct strategies enable learners to get access to another way to solve communication problems themselves and get across their intended meaning. These include most traditionally identified CSs such as message abandonment, approximation and code switching. Indirect strategies create conditions indirectly for interlocutors to reach mutual understanding, including stalling strategies such as use of fillers and repetitions. Indirect strategies are significant as they signal persistent effort rather than disengagement. Interactional strategies lead learners to co-construct their intended meaning, including the use of appeal for help, asking for repetition, asking for confirmation, expressing non-understanding and other strategies. Dörnyei and Scott (1997) further mapped these categories onto four types of communication problems (resource deficit, own-performance problems, other-performance problems and processing time pressure), forming a 3-by-4 matrix classification of CSs. Given its thoroughness, this model serves as the primary classification framework for the current study.

The classification research of CSs has gradually developed from the early simpler frameworks to more detailed classification systems, still further integration of affective and cultural factors is needed to refine CSs classification and enhance its applicability in language teaching (Cohen, 2014). One notable finding is that different cultures may cultivate different preferences in using CSs. For Chinese learners, specifically, the language distance between learners’ L1 and L2, as well as Chinese cultural factors, contributed to Chinese EFL learners’ particular preferences for certain CSs (Chen, 1990; Zhu et al., 2024).

### 2.2. Negotiation of Meaning

NoM refers to the collaborative work of speakers to achieve mutual understanding when there is an incomplete understanding in their communication (Ellis, 1994). NoM is described in two main ways: Varonis and Gass’s (1985) trigger-resolution model and Long’s (1983a, 1983b) model of three Cs—clarification request, confirmation checks and comprehension checks. Varonis and Gass (1985) build a two-part model to describe NoM: a trigger, which is the speaker’s utterance indicating non-understanding, and a resolution, which consists of an indicator of non-understanding by the hearer, followed by a response and a reaction by the speaker. Varonis and Gass’s (1985) model provides a detailed micro-analysis of repair mechanisms in communication breakdowns. In contrast, Long’s 3Cs focus on interactional modifications that pre-emptively facilitate comprehension. Specifically, clarification requests are expressions used by interlocutors to elicit clarification of the interlocutor’s preceding utterance. Confirmation checks are expressions used to confirm whether others have understood the utterance. Comprehension checks are expressions used to acknowledge whether others have understood the speaker’s preceding utterance(s). While both frameworks contribute to understanding NoM, this study adopts Long’s 3Cs due to its pedagogical applicability and effectiveness in analyzing meaning negotiation within task-based classroom interactions.

Previous research on NoM did not include other kinds of CSs. Samuda (2010) argued that early studies were overly focused on NoM, neglecting the fact that tasks can generate a significant amount of talk beyond NoM. García Mayo (2005) and Foster and Ohta (2005) noted that learners can actively use other strategies to assist one another, and that NoM is just one of many interaction processes that promote language development. In actual communication, speakers use not only negotiation devices to solve communication problems but also other CSs to enhance communication. It is therefore essential to investigate not only NoM but also other CSs used by learners to maintain and develop interaction in different contexts (Nakatani, 2010).

In addition, while both Varonis and Gass’s and Long’s models have significantly advanced our understanding of NoM, they predominantly focus on verbal interaction, overlooking the critical role of non-verbal cues—such as gestures, facial expressions, body language and tone of voice. Non-verbal cues are important as they often reinforce, complement or even contradict verbal interaction (Keelson et al., 2024). These cues are indispensable for conveying speakers’ emotions, attitudes and intentions, yet they remain understudied. Crucially, non-verbal communication is deeply intertwined with verbal interaction and intercultural differences can lead to miscommunication if unaddressed (Belío-Apaolaza & Muñoz, 2024). Given their importance to communicative dynamics (Ji et al., 2025), reliance on audio recordings alone risks omitting key aspects of NoM. To capture the full spectrum of meaning negotiation, video recordings are essential in empirical research, enabling analysis of both verbal and non-verbal dimensions.

In SLA, it is generally believed that more instances of NoM can lead to more second language development opportunities, therefore many studies have investigated how features of communicative tasks influence learners’ NoM (H. Wang, 2019). According to Doughty and Pica (1986), tasks can be classified into optional exchange tasks and required information exchange tasks. In optional exchange tasks, such as decision-making tasks, interlocutors share information and the task can be completed without information exchange. While in required information exchange tasks, such as information-gap tasks, interlocutors possess only pieces of information and must exchange information to finish the task (Yan et al., 2025). Doughty and Pica (1986) hypothesized that a required information exchange task may produce more NoM than an optional information exchange task as it requires more information exchange. However, the results of empirical research on this hypothesis are mixed (Yan et al., 2025). Newton (2013) argues that we need not only investigate the task-type effects on quantity (how much) of negotiation but also the qualitative aspects of NoM, including what is negotiated and how these interactions unfold.

While significant progress has been made in understanding CSs and NoM, several gaps remain. First, the role of CSs as a means of facilitating meaning negotiation has not been adequately investigated. Most previous studies have been quantitative and focused on formal language classrooms. However, since CSs and NoM are primarily used in interaction, a more nuanced and detailed examination of CSs and NoM should be conducted within natural contexts, with an emphasis on the interactional environment. Second, there is a lack of consensus on how different types of tasks affect the frequency and effectiveness of these strategies. Further investigation into the role of task design in promoting NoM and CSs use are needed. Third, the influence of cultural and contextual factors on the use of NoM and CSs requires further research. Most of the existing studies focus on Western contexts, neglecting the uniqueness of CSs and NoM in non-Western cultures. According to Hofstede (2011), there are significant differences between high-context cultures (such as China) and low-context cultures (such as the United States) in the choice of CSs. In high-context cultures, the message is exchanged more implicitly and indirectly, with much non-verbal coding (Broeder, 2021). However, existing studies have not fully explored the uniqueness of CSs and NoM in high-context cultures, especially the behaviors in Chinese EFL learners’ peer interactions. Therefore, the present study aims to investigate how Chinese EFL students use CSs to negotiate meaning with their peers during different communicative tasks in an intensive English language program. The research questions are:

(1) What communication strategies are used frequently by EFL learners to negotiate in their peer interaction during decision-making and information-gap tasks?
(2) How do EFL learners apply these communication strategies to facilitate meaning negotiation in peer interaction during these tasks?

## 3. Material and Methods

### 3.1. Setting and Participants

All participants were from an International Summer Intensive English Program, which was jointly organized by a double first-class university in China and a university in the United States. The program aimed to improve Chinese students’ oral English skills and enhance their ability to communicate effectively. The program lasted 5 weeks and involved 30 American ESL/EFL teachers and 900 Chinese EFL students. The students were divided into 30 classes, which are taught by the teachers in turn. The program provided students with an immersive English learning experience, which was quite different from the regular English classes in China, as it provided more opportunities for communication in English. The phenomenon of students continuing to communicate with each other in English even outside of class was evident throughout the program.

Sixteen students (eight females and eight males) and one native English-speaking teacher agreed to participate in this study. The students were all first-year non-English majors, with an average age of 20, and had been studying English for an average of 7 years. The teacher, a native English speaker from a university in the United States, was highly experienced in teaching English as a second and foreign language. She held a PhD in linguistics and was an associate professor, often encouraging students to actively participate in a variety of communicative activities.

### 3.2. The Tasks

Two tasks were implemented to observe students’ interactions (see Appendix A). The first task was a decision-making task in which students were asked to imagine themselves as travel agents and create a travel plan for their clients. This task was completed over two class periods (45 min per class), and students worked in groups of three. Students were required to make decisions about travel destinations, routes and scenery spots to visit, as well as address logistical details such as hotels, meals and time management.

The second task was an information-gap activity. Students worked in pairs and each pair was given two pictures that appeared quite similar but contained several differences. Without showing each other their own picture, students had to collaborate through communication to identify the differences. Both tasks were integrated into the normal class schedule and presented as part of the regular class routine. These tasks were meaning-focused, requiring students to exchange information and reach a conclusion.

### 3.3. Data Collection and Analysis

Data were collected through video recordings in the second week of the program since students were already familiar with each other and with the teacher at that time. The students were aware that they were being videotaped. However, the engaging and harmonious classroom environment, coupled with the teacher’s encouragement and established familiarity with the students, appeared to mitigate potential effects on their behavior, as evidenced by the recordings. Specifically, two groups of three students, each comprising two females and one male, were observed in the decision-making task, and one dyad (one female and one male) were observed in the information-gap task. All groups were randomly chosen during the group work tasks in the class. In all, the study collected 4.5 h of interaction (eleven videotaped sessions).

The discourse data were firstly transcribed (see Appendix B) and analyzed. Non-verbal communication is also transcribed since it is used as an important strategy by the speaker. The researcher went through all video clips three times and got the final transcription. For data analysis, the researcher first identified all episodes of NoM adopting Long’s three Cs criteria (Long, 1983a, 1983b). When encountering communication breakdowns, the students used confirmation checks, comprehension checks and clarification requests to make sure that their utterances were correctly understood by others or that they had correctly understood others. Frequencies of the three Cs were then counted.

Secondly, the transcripts were coded to identify the CSs that participants used in each episode of NoM. Building upon prior empirical results of CSs’ classification (Tarone, 1981; Færch & Kasper, 1984; Chen, 1990; Dörnyei & Scott, 1997; Foster & Ohta, 2005) and the data collected in this study, nine kinds of CSs were identified as coding category: repetition (R), rephrasing (Rp), restructuring (Rs), code switching (C-S), co-construction (C-C), message abandonment (MA), exemplification (E), appeal for help (AFH) and mime (M). Definition and elaboration of each category with examples from the data are presented in Table 1 as follows:

After all the CSs were identified, the frequency of each CS used in each group and the total amount and percentage of CSs were counted. Inter-rater reliability was checked for all transcriptions by inviting another rater to independently review all coding episodes of NoM and CSs. The researcher and the additional rater reached 90% agreement. Disagreements were solved by reviewing the definition and exemplification of each CS and discussion. The CS may appeared singly or embedded in another within an utterance. Salient instances of CSs were subsequently extracted and subjected to further analysis for in-depth discussion.

## 4. Results

### 4.1. Quantitative Results of Students’ Behavior in Peer Interactions

In peer interaction, 107 episodes of NoM were identified within a total of 851 units, constituting 12.57% of the total interaction. Descriptive results of NoM in peer interaction are listed in Table 2. Among the three groups, negotiation was most frequent in the third group, constituting 14.24% of their interactions. In terms of negotiation strategies, the use of confirmation checks prominently outnumbered that of comprehension checks and clarification request across all groups.

The students used various CSs to facilitate the NoM, amounting to a total of 469 CSs that were categorized into three broad categories encompassing nine distinct CSs. Table 3 provides a detailed description of the number and percentage of each CS, categorized by categorization and problem type. Direct strategies were the most frequently employed, constituting 54.16% of all CSs. For direct strategies, the students used code-switching (38.17%) and mime (5.12%) frequently. Indirect strategies followed, accounting for 39.87% of the CSs, and were primarily characterized by the repetition strategy. Interactional strategies accounted for only 5.97%, within which students used CSs of appeal for help (3.41%) and co-construction (2.56%).

### 4.2. Qualitative Results of Students’ Behavior in Peer Interactions

#### 4.2.1. Lexical and Content-Level Negotiation

In peer interaction, the students negotiated mostly at lexical and content levels. They did not pay much attention to form at the sentence level. Below are examples of the students’ negotiation at lexical and content levels.
**Excerpt 1: Negotiating meaning at the lexical level (Group 2)**99Lily: How to get to the Er hai lake? How to get there?100Dara:Take a bus, **we will take, we will, umm..游轮, 游轮 (yacht).** (R, C-S, AFH)101Lily: **Travel, travel ship**. (R)102Dara:First we take a bus to get there, and then we by a ship, no, it’s not the...103Sandy:**Ship, visiting ship.** (R)104Dara:**Visiting ship**, on the ship um we can see the lake, and then **Bai, Bai tripe, traip, they will**, we can watch their performance, they are singing and dancing. (R, S-R, Rs)

In this excerpt, the students were negotiating the word of “yacht”. Dara initially repeated part of her utterance, “we will”, in an effort to recall the English equivalent for the Chinese term “游轮”. Despite this attempt, she was unable to retrieve the correct term and resorted to using the Chinese word to request assistance from her peers. Lily responded with a literal translation, rendering “游轮” as “travel ship”. Dara then reformulated her previous statement, but soon recognized that Lily’s translation did not accurately capture her intended meaning, leading her to directly dismiss it with “no”. The boy Sandy then offered an alternative translation, “visiting ship”, which Dara repeated and continued her talk. At Line 104, Dara attempted to articulate “Bai tribe” but mistakenly pronounced it as “tripe.” Sensing that it might be wrong, she attempted a self-repair, adjusting the pronunciation to “traip”, which remained incorrect. However, her peers did not intervene to correct the mispronunciation and Dara proceeded with the conversation.
**Excerpt 2: Negotiating meaning at the content level (Group 1)**79Nancy: I have questions, so **our, our** advertisements are judged **most, mostly** Chinese or foreigners? (R, S-R)80Andy:Ummm, which…81Nancy:**就是我们的广告是针对中国的还是针对外国的? (That is to say whether our advertisement is target at home or abroad?)** (C-S)82Andy: Yes (pointing to Nancy), a good question!

 (All laughing)83Anna:I think both Chinese and foreigners.84Andy:Chinese…**if, if**… (R)85Nancy:I mean, **if, if** we want to attract the foreigners, we have to choose **like** umm. Tian an men square, something like that. But if it’s about Chinese, we have choose more nature points. (R, E)86Andy:Yes, yes. (R)87Anna: (Turn to Nancy) **You decide it, you decide it**. (R)

In this conversation, the students were engaged in the task of selecting a travel destination. Nancy proposed that they need to first make sure whether their customers will be Chinese or foreigners. This proposition was deemed significant by her peers. At Line 79, Nancy repeated the word “our” showing that she was thinking about how to express the question. She also repaired herself by using the adverbial form of “most”, yet the resulting sentence remained grammatically incorrect. In Line 80, Andy stalled his words showing that he did not understand it. Consequently, in Line 81, Nancy opted to convey her question directly in Chinese. At Line 85, Nancy elucidated her point further by providing examples, which facilitated a clear understanding among the other participants. Both Andy and Anna reiterated their responses affirming their grasp of Nancy’s proposal.
**Excerpt 3: Negotiating meaning at the lexical and content level (Group 1)**29Andy: **I think it’s… I know it‘s, ummm** Hawaii. (Rs)30Nancy:**Hawaii?** (Shaking head) No. 31Anna:**Hawaii**… (R)32Andy:No?… You? (Turn to Anna who is thinking) **This, this one (Drawing on the paper) 美国的这边…阿拉斯加. (On this side of America…Alaska)** (M, C-S)33Anna& Nancy:**Alaska.** (O-R)34Andy:**Alaska.** (R)35Nancy:Yeah … Alaska is too cold.36Andy:**Spend. Spend** summer vacation…summer vacation? (Writing on the paper) Place, time and who. (R)37Anna:**Who?**38Andy:Where, when, who.39Anna:**But we are doing... we are travel agencies**, we plan this trip and… (Rs)40Nancy:…**just for those people**. (C-C)41Anna:Yeah, people can come here and choose which place they want to go, so there’s no ‘who’. **Understand me?** (Turn to Andy) **Understand me?** (R)42Andy: I beg your pardon? (Laughing) I know I know. Ok, **place, place, which state? Which state you see in America?** (R, Rp)

In this excerpt, the students were trying to reach an agreement about the optimal travel destination for their clients. Andy initially suggested Hawaii but was rejected by Nancy. Subsequently, Andy attempted to propose Alaska; however, he encountered difficulties in articulating the name in English. To overcome this, he resorted to drawing a pictorial representation of the location and concurrently provided the Chinese term at Line 32. The other participants promptly grasped his intent and spontaneously offered the English equivalent, which Andy then echoed. Following Nancy’s objection that Alaska was too cold, Andy countered by suggesting it as a potential destination for summer vacations. He subsequently summarized the key considerations they needed to address.

At Line 37, Anna voiced her skepticism regarding the “who” component with a confirmatory query, indicating her belief that this aspect was superfluous. In Line 39, she attempted to construct a sentence illustrating her meaning but struggled to complete it, leading her to restructure her statement. In Line 40, Nancy completed Anna’s unfinished sentence and was confirmed by Anna. Anna then tried to confirm if the boy has understood her by repeating “do you understand me?” in Line 41. Andy joked by pretending not understanding the girls’ words and then confirmed by repeating “I know”.

#### 4.2.2. Self-Repair and Other-Repair

The findings revealed a higher incidence of self-repair compared to other-repair in students’ interactions. As presented in Excerpt 1 (Line 104) and Excerpt 2 (Line 79), the students repaired their own errors to produce more accurate expressions even though these errors did not cause communication failure. As for other-repair, the frequency was notably low. Students repaired their peers’ mistakes mostly on lexis (e.g., Line 33 of Excerpt 3) and content level (e.g., Excerpt 4). Direct correction of grammatical mistakes in peers’ utterances was avoided, as demonstrated in Excerpt 5. The sole instance where students explicitly pointed out their peers’ errors pertained to pronunciation, as detailed in Excerpt 6.
**Excerpt 4:**91Nancy: What else? … So **the second day**...92Andy:**Third day.**93Nancy: **Oh, no, the third day**, what, where...
**Excerpt 5:**5Brutes: And uh you note **how many, how much stones**?6Della:**How many stones**?7Brutes: Yes.
**Excerpt 6:**203Brutes: I don’t know, but I know **I’m cau(si)ous.**204Della:**Cautious.**205Brutes: Yes.

#### 4.2.3. Repetition

The result shows that repetition was used most frequently by the students. Repetition was found to serve three distinct functions: (1) to stall and gain time for thought; (2) to provide the listener with another chance to hear and process the information; and (3) to emphasize certain messages and convey emotions. Examples of self-repetition illustrating each function are presented below:
**Excerpt 7: Repetition to stall and gain time to think**98Nancy: =Henan has **a lot of, a lot of, umm, a lot of, umm**, attractings about human. 
**Excerpt 8: Repetition to provide the listener another chance to hear**161Nancy:(Pointing to the map on the paper) **Which city** are there? **Which city?**
**Excerpt 9: Repetition to emphasize and show emotions**85Nancy: I mean, if, if we want to attract the foreigners, we have to choose like, umm, Tian an men square, something like that. But if it’s about Chinese, we have choose more nature points.86Andy:**Yes, yes.**87Anna:(Turn to Nancy) **You decide it, you decide it.**

When peers proposed significant ideas or provided words or messages they wished to convey, the students tended to employ repetition, particularly using utterances such as “yes” or “no” to demonstrate strong affirmation or rejection. Sometimes, the three functions of repetition were observed to occur simultaneously. Out of the total 154 instances of self-repetition in peer interactions, 99 instances were attributed to the first function, 31 to the second and 24 to the third.

#### 4.2.4. Code-Switching

The students also frequently resorted to their L1, with code-switching CS constituting 38.17% of their interactions. This reliance on L1 occurred particularly when they encountered difficulties in expressing certain meanings in English, such as with proper nouns for places, exemplified by the use of “好莱坞” instead of “Hollywood”. Additionally, they employed L1 for specific terms that were difficult to translate or articulate in English, as illustrated in Excerpt 10.
**Excerpt 10:**117Andy:First we can go to Jinan to see the Mount Tai, do you know Mount Tai? **The泰斗(most famous) of the five mountain.** (Rp, C-S)118Nancy: Oh, oh.119Anna: There is a Baotu**泉 (spring)**… (C-S)

Secondly, the students resorted to their L1 when they became impatient and were disinclined to elaborate further on their points, as evidenced in Line 140 of Excerpt 11 (Nancy: 就是大衣**That is coat**) and Line 175 of Excerpt 12 (Nancy: 就是啤酒节嘛**That is beer festival**). Similarly, they employed L1 to convey information swiftly when their peers exhibited impatience, as illustrated in Line 183 of Excerpt 12 (Andy: 八月中旬哦**In the middle of August**). The use of L1 by one participant could often prompt others to follow suit, as demonstrated in Excerpt 12 (Lines 183–184). At times, L1 was employed instinctively to bridge conversational breakdowns and silences.
**Excerpt 11:**137Andy:Umm, we, we will spend a day on it so at midnight we will get off and then have a nap.138Anna:So where? In hotel? Go camping? In a camp?139Andy: No, no camp, but sit in the seat and you can rent a coat. **(Drawing a coat on the paper)**140Nancy:**就是大衣. (That is coat)**141Andy: Yes, yes, wear a coat and wait for the sunrise.

In Excerpt 11, the boy Andy suggested that they can spend a day on Mount Tai. At Line 139, he mentioned that they can rent a coat. Anticipating potential misunderstanding from his peers, he attempted to draw a coat on paper to clarify his point. Subsequently, at Line 140, Nancy became a bit impatient and opted to use Chinese directly to convey her understanding of Andy’s meaning.
**Excerpt 12:**173Nancy:Is Qingdao has a festival? **Festival.** (R)174Andy:Festival... (R) 175Nancy:**就是啤酒节嘛. (That is beer festival)** (C-S)176Andy:Yes, it’s at **Aogt...**177Anna& Nancy:**August.** (O-R)
178Andy:**August.** (R)179Nancy: **We can, we can** make a trip to the festival. (R)180Anna:Well, yes.181Andy:**The, the** middle of the… (R)182Anna:**Okay, okay.**183Andy:**八月中旬哦. (In the middle of August)** (C-S)184Nancy: **正好八月中旬去看海. (We can also see the sea at that time)** (C-S)185Andy:Yes, ok, you can go to the **sea, seaside.** (S-R)

In Excerpt 12, Nancy asked about the beer festival in Qingdao, repeating the word “festival” to offer her peers an additional opportunity to comprehend her query. However, Andy failed to grasp her meaning, as he repeated the word to stall. Consequently, Nancy directly provided the term in Chinese. At Line 176, Andy attempted to point out the specific time of the festival, but incorrectly pronounced “August” as “Aogt”. The two girls then spontaneously corrected his pronunciation to “August”, which Andy subsequently repeated. In Line 181, Andy struggled again with the pronunciation of “August”, prompting Anna to interrupt him impatiently by repeating “okay”. Andy quickly resorted to Chinese, which led to Nancy’s response in the same language. Andy subsequently confirmed Nancy’s suggestion by repeating and translating her sentence into English. Throughout the dialogue, Andy endeavored to utilize English, notwithstanding the challenges he encountered.

#### 4.2.5. Mime

In addition to verbal communication, the students also employed a variety of non-verbal CSs effectively. When unable to articulate actions in English, students utilized gestures as demonstrated in Excerpt 13. Similarly, they used gestures to depict the shape of objects, as illustrated in Excerpt 14 (Lines 83–97). Additionally, students resorted to drawing to communicate their intentions, such as in Excerpt 3 (Line 32, where a map was drawn) and Excerpt 11 (Line 139, where a coat was sketched).
**Excerpt 13:**119Anna: There is a Baotu泉 (spring)… (C-S)120Andy: Yea, it is in Jinan.121Anna:Is it still… **(Making the gesture of spraying)**? (M)122Andy:Yeah, **yes, yes, yes.** (R) 

In Excerpt 13, the students negotiated meaning successfully through non-verbal strategy. In Line 121, Anna inquired whether Baotu Spring was still “spraying.” Struggling to recall the word “spray,” she demonstrated the action by mimicking the motion of water springing up. Recognizing her reference, Andy responded affirmatively by repeating “yes” three times to emphasize his strong agreement.
**Excerpt 14:**81Della: **And there is... The tree up is, umm, the body of the tree, the body of the tree**, what’s its shape? (MA, R)82Brutes: **Shape, umm.** (R)83Della: **Shape, like this (Making gesture)... the body of the tree, the body of the tree. (M, R)**84Brutes: **The body of the tree...** (R)85Della:**Body, body, body, body of the tree**, what’s the shape **of his, of its body?** (R, S-R)86Brutes:Umm, I don’t know how to say.87Della:**(Making gesture) Like this or?** (M)88Brutes:**(Making gesture of a bight) 曲线** (M, C-S)89Della:**Like this (Making gesture)?** Yeah? Or on one... (M)90Brutes: **Like this (Making gesture).** (M)91Della:This? Oh, this, so...=92Brutes: =**Do, do** you refer to, umm, the tree... (R)93Della:**The, the, on the left, on the left side, the branches is like this, the branches like this, right? (Making gesture of a half circle)** (R, M)94Brutes: Yeah.95Della:Yes, and in the middle, t**he branches like this. (Both W and D makes gesture of straight)** (M)96Brutes:Yeah.97Della: **And on the right side like this. (Both W and D makes gesture on the right side)** (M)98Brutes: Yeah.99Della: Yes, yes, so it’s the same.

This is a typical example showing how learners used non-verbal strategy effectively. At Line 81, Della asked about the shape of the tree’s trunk. Following the boy’s hesitation, she tried to clarify her meaning again in Line 83, employing both verbal and non-verbal CSs. Despite this, Brutes remained unclear about her intent. Consequently, in Line 85, Della repeated the word “body” for four times to emphasize. Brutes eventually grasped her meaning but verbally indicated his inability to express it in words. Between Lines 87 and 90, Della prompted Brutes to utilize gestures to communicate. However, Della’s question regarding the tree’s trunk shape was still ambiguous, leading Brutes to seek confirmation in Line 92. Della, getting a bit anxious, clarified her query more explicitly through more exaggerated gestures and multiple repetitions. Brutes ultimately confirmed Della’s description. Recognizing the success of this non-verbal approach, then from Line 93 to 98, they used gestures collaboratively to figure out the shape of the tree on their pictures and swiftly arrived at a consensus. 

#### 4.2.6. Co-Construction and Appeal for Help

The students also utilized co-construction and appeal for help CSs to support one another in peer interaction. Co-construction CSs were employed when one interlocutor hesitated on an utterance and the partner provided suitable words or phrases to complete the missing element. All co-construction CSs provided by the students were suggestions, focusing on content and lexical elements rather than linguistic items, as demonstrated in Excerpts 15–17.
**Excerpt 15:**128Anna:**We can go there…**129Andy: …**and making a night!**130Nancy:You mean, you mean spend a night on Mount Tai?131Andy:Yeah.
**Excerpt 16:**213Andy: Yeah, it’s in the…**so we can…**214Anna: **Take a boat!**215Andy: **Yeah! It’s about one hour.**
**Excerpt 17:**51Lily:Then how to **get to, get to, to, to...**52Dara: **Dalian**?53Lily: **No, no, no, uh-mm, Yushi?**

When encountering difficulties during peer interaction, the students sought assistance from their peers through direct or indirect appeals for help, predominantly concerning lexical items. As illustrated in Excerpts 18 and 19, they utilized L1 to inquire “How to say or spell (a Chinese word) in English”. In response, their peers endeavored to provide assistance. Even in instances where peers were uncertain, they still offered feedback, contributing to the collaborative resolution of the communication challenge.
**Excerpt 18:**21Dara: … The spring time, umm, I mean there, (write on the paper) mild, **温和, 怎么拼?(How to spell mild?)**22Lily: **Mild, m-i-l-d.**23Dara: Mild.
**Excerpt 19:**128Brutes:Finish the line, and, umm, **大写怎么说(how to say bold), =bold?**129Della:Yes, yes, in, in …=**bold, bold? Yes, yes, bold, ok, ok, it’s fine.**

## 5. Discussion

### 5.1. Frequently Used CSs to Negotiate in Different Tasks

For research question one, quantitative results showed that negotiation was more frequent in the information-gap task than the decision-making task. This aligns with the results of prior research, which suggests that controlled and goal-convergent interactions generate more NoM than those less structured and open-ended interactions do (Nakahama et al., 2001; Yan et al., 2025). It also proved Doughty and Pica’s (1986) hypothesis that required information exchange task (i.e., information-gap task) may produce more NoM than optional information exchange task (i.e., decision-making task). As discussed in the literature review, an information-gap task is different from a decision-making task as it requires information exchange. The decision-making task had no standard answer and students may omit or avoid information exchanges that pose communication challenges, while in the information-gap task, students were required to identify differences and similarities between their respective pictures. This requirement necessitates information exchange about whether items were identical or distinct, which leads to a higher incidence of negotiation.

For negotiation strategies, confirmation checks were used much more frequently than comprehension checks and clarification requests, especially in the information-gap task. This finding diverge from previous studies reporting that students rarely use confirmation checks (McDonough et al., 2023; Xu & Shu, 2024). This difference may be due to the different tasks involved, as tasks employed in these studies were more open-ended, without mandatory outcome requirements, which may lead students to ignore non-understanding (McDonough et al., 2023), whereas tasks in this study, especially the information-gap task, necessitate concrete student output, which may have increased students’ remedial engagement demands. In addition, compared to other strategies, confirmation checks typically necessitate a simple responses without demanding additional information from interlocutors, representing an economic way to require assistance (Chang & Liu, 2016).

For CSs used to negotiate, direct strategies were the most frequently employed, followed by indirect strategies. This indicates a tendency among students to opt for alternative, self-contained methods to convey meaning. This finding runs counter to Chang and Liu’s (2016) observation that Chinese English speakers used more indirect strategies than direct strategies. This discrepancy may stem from the design of tasks, as tasks in this study required students to complete without time limitation, while tasks in Chang and Liu’s (2016) were time-limited. In time-pressured tasks, learners use more indirect strategies to mediate thinking process and maintain the conversation (Chang & Liu, 2016). Aligned with Chang and Liu’s findings (2016), interactional strategies were less frequently used, indicating that learners preferred to count on themselves to solve communication problems. Collectively, the findings indicated that the students can employ a variety of CSs to resolve communication problems rather than avoiding or ignoring them.

### 5.2. Learners’ Behaviors in Applying CSs to Facilitate NoM

In peer interaction, the students tried their best to get their meaning across and keep the conversations moving forward. Their negotiations primarily occurred at lexical and content levels, and students initiated more self-repair than other-repair. This aligns with most previous studies showing that speakers are more likely to use self-initiated repair than other-initiated repair as listeners assume that the speaker will repair themselves (Schegloff et al., 1977; García Mayo, 2005). The students repaired their own errors mainly to enhance accuracy (Jamshidnejad, 2011). Other-repair occurred less frequently. This may be attributed to several factors. First, as the purposes of the tasks are to communicate to finish tasks rather than achieve perfect English, correcting grammar and pronunciation do not help add more information to the conversation. Second, the students may be inclined to avoid face-threatening acts and to maintain an equal status among peers (Chang & Liu, 2016). Additionally, it may stem from the students’ limited knowledge and proficiency, which hinders their ability to recognize and address their peers’ mistakes effectively.

Consistent with previous studies’ findings (Lai & Wang, 2010; D. Wang et al., 2015), the students used more achievement strategies than reduction strategies, suggesting that Chinese learners are perseverant and achievement-motivated (D. Wang et al., 2015). The students employed various CSs when faced with challenges, resorting to abandonment strategy only when no other solutions are apparent. For these non-English majors, opportunities to communicate in English, particularly with native speakers, are rare. The program offered these students a contact with target-language communities, which may have stimulated their motivation and interest to communicate in English (Sun et al., 2022). 

Overall, the students used repetition and code-switching CSs more frequently. Previous research shows that repetition enables the speaker to stall for time and offers the listener additional opportunities to hear and process the information (Tarone & Yule, 1987). This study found an additional function of repetition, which is to emphasize certain messages and convey emotions. Rather than abandoning their intended expressions, the students resorted to self-repetition, which demonstrated their achievement motivation but also suggested inflexibility in their use of CSs (D. Wang et al., 2015). The students also frequently used their L1. Code-switching is particularly useful when students face production problems and need to maintain the flow of conversation (Shabani et al., 2022). This study also found the emotional function of code-switching, as students used L1 when they or when their peers became impatient. Furthermore, contrary to previous studies that suggested that Chinese learners may avoid the use of non-verbal CSs due to perceptions of impoliteness in Chinese culture (Chen, 1990; Lai & Wang, 2010; D. Wang et al., 2015), this study found that students employed non-verbal CSs effectively. This may reflect changes in China’s higher education from traditional learning to more task-based learning. In task-based learning, students pay more attention to the completion of tasks and may not think too much about interpersonal relationships (Fang, 2024). In addition, the students utilized co-construction and appealed for help with CSs, indicating their capacity to provide mutual assistance during interactions, either by directly requesting help from peers or by co-constructing their peers’ utterances to bridge understanding gaps (García Mayo, 2005).

## 6. Conclusions

In conclusion, the study found that negotiation occurs more frequently in information-gap tasks than decision-making tasks, with students primarily employing confirmation checks to signal communication breakdowns. For CSs used to negotiate, direct strategies were employed most frequently, in which students mainly used code-switch and mime. Indirect strategies followed, with repetition occur as the most frequently employed CSs. Interactional strategies, including co-construction and appeal for help CSs, were less frequently used. 

This study carries some implications for language instruction. While students possess the inherent ability to effectively utilize CSs, they often lack a structured understanding and knowledge in these strategies. To cultivate such knowledge, teachers should first raise their own awareness and that of their students by incorporating CSs into the class. It is essential to provide students with a comprehensive understanding of the advantages and disadvantages associated with each type of CS. For example, although repetition can sustain the flow of communication and prevent awkward silences, an overreliance on it may lead to interruptions and provoke impatience among listeners. Furthermore, teachers should design diverse communication tasks to improve classroom atmosphere and boost students’ engagement and motivation (Guo et al., 2025).

## Figures and Tables

**Table 1:** Categories of CSs.

Communication Strategies	Definition	Examples from the Transcription
Repetition (R) (Self-repetition, S-R; Other repetition, O-R).	Repeating a word or words by oneself or by the interlocutor immediately after they were said. Subclassified into self-repetition and other-repetition.	**On the right, on the right** have a tree. (S-R) --What’s your **birthday**? **--Birthday**, umm…(O-R)
Rephrasing (Rp)	Rephrasing the previous utterance by adding something or using paraphrase.	You in summer? **Are you born in summer?**
Restructuring (Rs)	Abandoning the execution of a verbal plan because of language difficulties and expressing the intended message in another way.	It can, um, **make**, um, **practice** my listening and **may, I may** study some new words from the movies.
Code switching (C-S)	Using native language term directly in L2 speech.	And then we can go along the **海岸线 (coastline)**
Co-construction (C-C)	One person completes what another has begun or various people create an utterance together.	--Ok, so this is Qingdao **and we can…** --**go beaches and beer festival**
Message abandonment (MA)	Leaving a message unfinished because one finds it difficult to continue due to language difficulty.	**He can use some simple examples to…** and then if we can…
Exemplification (E)	Using examples to illustrate one’s intended meaning.	I don’t know what is the Chinese, but that’s a place where you can see how they make films **like birds play or volcano eruption, or a car crash**…how they make films!
Appeal for help (AFH)	Turning to the interlocutor for help directly or indirectly due to a gap in one’s L2 knowledge.	Um, look up in the dictionary, **how to say **“接机** (airport pickup)”**
Mime (M)	Describing a concept nonverbally, or accompanying a verbal strategy with a visual illustration.	--There is a Baotu quan… --Yeah, it is in Jinan --**Is it still…(making the gesture of springing up)?** --Yeah, yes, yes, yes

**Table 2:** Number and percentages of each type of NoM across all interactions.

NoM	Group 1	Group 2	Group 3	Total
Total units of interactions	317	218	316	851
Units of confirmation checks	16	12	30	58
Units of comprehension checks	8	6	10	24
Units of clarification request	12	8	5	25
Percentage of units of NoM/total units of interaction	11.36%	11.93%	14.24%	12.57%

**Table 3:** Number and percentage of CSs used by participants in all instances of NoM (*n* = 469).

Category	Problem Type	CS	*n* (Percentage)
Direct strategies	Resource-deficit	Message abandonment (MA)	8 (1.70%)
		Code switching (C-S)	179 (38.17%)
		Mime (M)	24 (5.12%)
		Exemplification (E)	6 (1.28%)
		Restructuring (Rs)	15 (3.20%)
	Own-performance	Rephrasing (Rp)	22 (4.69%)
Interactional strategies	Resource-deficit	Appeal for help (AFH)	16 (3.41%)
	Other-performance	Co-construction (C-C)	12 (2.56%)
Indirect strategies	Processing time	Repetition (R)	187 (39.87%)

## Data Availability

The original contributions presented in this study are included in the article. Further inquiries can be directed to the author.

## Appendix A

### Appendix A.1. The Tasks

#### Appendix A.1.1. Task One: Decision-Making Task

In a group of three, imagine yourselves as a travel agency. Now you need to make a travel plan for your customers; you will first choose where to travel for your customers and then detailed schedule should be made out. You have to make a detailed plan like the route of your travel, how do you get there, where do you live, and what do you eat, how many days you will stay, etc. In the first class, you will come out with a general plan and after the class you will go and check the internet to collect detailed information that will be discussed in the second class. Make your plan into a picture, draw out your route, scenery spots, food, hotel, etc. and bring it to the next class.

#### Appendix A.1.2. Task Two: Information-Gap Task

In a group of two, each one of you is given a picture; there are several differences between your pictures. Do not show your picture to others and try to find out the different places in your pictures.

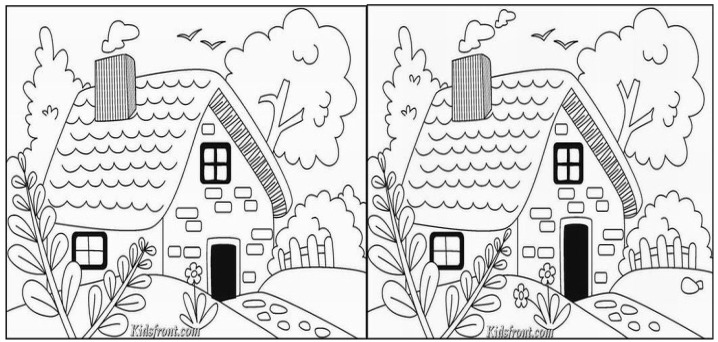

## Appendix B

### Transcription Conventions

=	Equal mark means this line overlaps with the next speaker’s utterance
…	Three dots indicates silence under 10 s
……	Six dots indicate silence beyond 10 s
$ $	Dollar mark means speaking while laughing
**Boldface**	Boldfaced places are words stressed to emphasize
[ ]	Words in square brackets are utterances uttered by the other interlocutor
(?)	Question mark in a bracket indicates words that cannot be identified
(Action)	Actions described in a bracket are paralinguistic transcriptions
